# Notes on Preparations for the Sick

**Published:** 1892-06

**Authors:** 


### Notes on Preparations for the Sick.

---

**Antiseptic Soap, containing Biniodide of Mercury (5 per cent.).**—Edward Cook & Co., Bow, London.

Until quite recently a satisfactory soap, containing as an antiseptic one of the salts of mercury, has been difficult to prepare, on account of the alkaline soap refusing to yield a good lather, oleate of mercury being formed—a compound which has little or no germicidal action. One of the most powerful antiseptics of the mercury salts is, as is well known, the bichloride. Moreover, it is cheap, and easily soluble; but it has the disadvantages of being extremely poisonous, and easily reduced by albuminoid matter, with which it combines, thus being rendered inactive. In a paper recently read before the Society of Chemical Industry in Glasgow, by John Thomson, the solubility of the red biniodide of mercury (which is claimed to be even a more powerful antiseptic than the bichloride) in iodide of potassium has been made use of. A soap can thus be easily prepared containing a certain proportion of the biniodide in a soluble form. It is stated to be permanent, having no tendency to separate, and to be more germicidal in its properties than any other antiseptic soap yet known. This soap has been used with much success in the treatment of chronic forms of eczema, also in parasitic skin diseases, and as a disinfectant in convalescence from scarlet fever.

---

**Nestlé's Milk Food.**—H. Nestlé, London.

The leading idea of the manufacturer of this food has been to combine Swiss milk of the very best quality with a substance which, while useful to the child as an aliment, would prevent the coagulation of the casein in large lumps. After many trials, M. Nestlé arrived at the conclusion that thoroughly baked wheat flour was the best adjunct to the milk, and an experience of twenty years has shown that he was right. The mixture of wheat flour, prepared according to an improved method, with carefully concentrated milk, forms a highly nutritious, palatable, and digestible powder, which can be kept for a long time, and sent to any part of the world without losing any of its properties. During the process of baking, the starch contained in the wheat flour is broken up and partly transformed into dextrine, while the gluten is rendered soluble.

We hold that prepared foods of this kind are infinitely safer for infant feeding in towns than even employing wet nurses or fresh cow's milk.

The prepared food has been efficiently sterilised, everything deleterious having been destroyed by cooking.

---

**Automatic Disinfector.**—GEORGE TAYLOR & CO., Liverpool.

The disinfector has simply to be placed in the ordinary w.c. cistern, without fixing or adjustment of any kind whatever. It consists of an ordinary bottle, fitted with a special discharging arrangement, by which a small quantity of the contained disinfectant is automatically administered at each flush. The apparatus works admirably as described, and cannot fail to be of service wherever there is need for constant disinfection.

---

**Hayden's Viburnum Compound.**—NEW YORK PHARMACEUTICAL COMPANY, London.

Yet another uterine tonic, said to be "the remedy *par excellence*." It is free from all narcotics, leaves no unpleasant effects, and is perfectly safe. It is said to be a combination of the active principles of the viburnum opulus, dioscorea villosa, and scutellaria lateriflora, combined with aromatics. It has much evidence in its favour, and is well worthy of a trial in all cases of dysmenorrhœa.

---

**Aerated Waters.**—PACKHAM & CO., Croydon.

These aerated and mineral waters are all prepared with distilled water, and are therefore likely to be free from anything pernicious.

---

**"Perfect Non Plus Ultra" Thermometer.** FERRIS & COMPANY, Bristol.

Messrs. Ferris & Co. have sent us a thermometer which, under this somewhat lengthy name, they have recently issued. It combines the advantages of their "Perfect" Thermometer with those of Mr. Hicks's "Non Plus Ultra," and should therefore become popular with the profession as a pocket companion.

